# Morpho-physiological growth performance and phytoremediation capabilities of selected xerophyte grass species toward Cr and Pb stress

**DOI:** 10.3389/fpls.2022.997120

**Published:** 2022-09-08

**Authors:** Taimoor Hassan Farooq, Muhammad Rafay, Hamza Basit, Awais Shakoor, Rubab Shabbir, Muhammad Umair Riaz, Baber Ali, Uttam Kumar, Kamal Ahmad Qureshi, Mariusz Jaremko

**Affiliations:** ^1^Bangor College China, A Joint Unit of Bangor University and Central South University of Forestry and Technology, Changsha, China; ^2^Department of Forestry, Range and Wildlife Management, Faculty of Agriculture, The Islamia University of Bahawalpur, Bahawalpur, Pakistan; ^3^Teagasc, Environment, Soils and Land Use Department, Johnstown Castle, Co., Wexford, Ireland; ^4^Department of Plant Breeding and Genetics, Seed Science and Technology, University of Agriculture, Faisalabad, Pakistan; ^5^Department of Plant Sciences, Quaid-i-Azam University, Islamabad, Pakistan; ^6^College of Plant Protection, Fujian Agriculture and Forestry University, Fuzhou, China; ^7^Department of Pharmaceutics, Unaizah College of Pharmacy, Qassim University, Unaizah, Saudi Arabia; ^8^Division of Biological and Environmental Sciences and Engineering, Smart-Health Initiative and Red Sea Research Center, King Abdullah University of Science and Technology, Thuwal, Saudi Arabia

**Keywords:** productivity, biochemical attributes, gas exchange, heavy metals, dry climate, Cholistan

## Abstract

Being sessile organisms, plants cannot escape unwanted changes in the environment. The rapid human population explosion caused significant environmental problems. Heavy metals produced through various sources can cause severe damage to living organisms. The study was planned to evaluate four grass species’ morpho-physiological growth characteristics and phytoremediation capabilities under chromium (Cr) and lead stress (Pb) in the arid climate. *Typha angustifolia*, *Tragus roxburghii*, *Aeluropus logopoides*, and *Cenchrus ciliaris* grass species were used for the study. One-year-old stubbles from the Cholistan desert were used for the experiment. Cr treatments in the form of K_2_Cr_2_O_7_ were applied at 0, 20, 40, and 80 mg L^–1^, whereas Pb was applied as PbCl_2_ at 0, 50, 200, and 500 mg L^–1^ as control, low, moderate and high-stress, respectively. After 6 weeks of heavy metals treatments, plants were harvested and analyzed for growth performance and phytoremediation capabilities. Results depicted that, regarding morphological attributes, *T. angustifolia* performed better, followed by *C. ciliaris*; no clear pattern was observed for *T. roxburghii* and *A. logopoides*. The CO_2_ assimilation rate (Co_2_d) and water use efficiency (WUE) increased as the heavy metal stress increased in all species under both metals. In contrast, total chlorophyll content was higher under low stress. Other physiological parameters, such as relative humidity (RHd), net photosynthetic rate (A), transpiration rate (E), stomatal conductance (Gs), leaf internal CO2 concentration (Ci) and membrane stability index (MSI) gradually decreased as the Cr, and Pb stress levels increased among all the species. Moreover, Cr and Pb absorption contents of *T. angustifolia* were higher than the other three species at each stress level. Overall, *T. angustifolia* thrived against heavy metals stress and showed higher biomass, maximum photosynthetic measurements, WUE and higher metal absorption among all the selected species. Results concluded that although all the selected species behaved fine under stress conditions, *T. angustifolia* performance was better; thus, it can be used to remediate the soil near industrial estates.

## Introduction

Abiotic stresses are assessed to be the main cause of the yield reduction in plants and thus, are considered a prodigious constraint to crop productivity (Yousaf et al., 2018; Gilani et al., 2020; Ali et al., 2021; Ahmad et al., 2022; Hussain et al., 2022; Nawaz et al., 2022; Panikar et al., 2022). Plants are often sensitive to the low and high accessibility of different heavy metal ions as essential micronutrients (Awad et al., 2021; Javed et al., 2021; Koleva et al., 2022; Shabbir et al., 2022; Xiao et al., 2022). Heavy metals are elements with a large atomic mass and are at least five times denser than water (Tchounwou et al., 2012; Singhal et al., 2022). They are not biodegradable in natural systems and are ready to mount health dangers when they exceed permissible concentrations (Javed et al., 2020; De Conti et al., 2021; Abbas et al., 2022). The key sources of heavy metal emissions are industries such as mining, painting, car-making, smelters, and metal refining (Singh et al., 2011). Rapid population increase, excessive industrialization, and challenging farming practices contribute to significant environmental problems and large quantities of hazardous waste being generated and disposed of in the soil (Lin et al., 2022). Heavy metals like lead (Pb), arsenic (As), cadmium (Cd), copper (Cu), zinc (Zn), nickel (Ni), chromium (Cr), and mercury (Hg) are continuously being introduced into the atmosphere by removing metropolitan sludge and industrial waste from agricultural and agrochemical soils, are the main components of inorganic pollutants (Gunatilake, 2015; Javad et al., 2022).

In recent years, compelling work into emerging cost-effective remediation techniques has contributed to public concerns about human health and environmental bullying resulting from soil contamination. The basic methods to cure polluted soils are cost-effective to install and maintain, namely chemical, physical and microbiological processes. A new green technique called phytoremediation has been developed, which uses plants to clean up soil, water, and the environment (Saha et al., 2017). Phytoremediation is dirt-free, uncomplicated, economical, and environmentally friendly, and the by-products will most notably have a range of other applications (De Conti et al., 2021). However, many restrictions on phytoremediation for pollution management involve a further study of plant and site-specific soil conditions. Environmental factors also decide phytoremediation’s effectiveness, as plants’ survival and development are badly affected by the harsh environment, toxicity, and soil characteristics in contaminated areas. Eventually, various forms of soil and water contaminants need different kinds of plant remediation vegetation, which adds to the complexity of the technology and requires a wide range of research activities before specific plants are released to commercialization (De Conti et al., 2021).

Due to their widespread toxicity, Cr and Pb, among heavy metals, have become more seriously toxic to plant functions (Nawaz et al., 2021; Shah et al., 2022). High soil Cr exposure causes oxidizing damage to plants, resulting in reactive oxygen species (ROS) formation by removing iron (Fe) from proteins and inhibiting the chloroplast and mitochondrial electron transport chains in plants (Scandalios, 2005). In addition, toxicity also influences the inhibition of the photosynthesis system and photosynthetic transportation of electrons (Romero-Puertas et al., 2007). Pb is not biodegradable in agricultural fields and can remain in the environment for 150–5,000 years (Saxena et al., 1999). Therefore, it is almost impossible to extract Pb from the soil once it reaches the soil system. It has a growth-inhibitor effect in plants, influencing photosynthesis and oxidative stress physiological and metabolic functions (Nouman et al., 2020).

Heavy metals (essential and non-essential) usually produce common toxic effects on plants, such as inhibition of growth, low biomass accumulation, photosynthesis reduction, senescence, altered water balance, and nutrient assimilation, which ultimately cause plant death (Singh et al., 2016). Therefore, this research is driven by the hope of decreasing the entry of heavy metals into crop plants, reducing the risk of contamination in animals and humans. The study’s objectives were to (1) evaluate four grass species (*Typha angustifolia*, *Tragus roxburghii*, *Aeluropus logopoides* and *Cenchrus ciliaris*) morpho-physiological growth characteristics and (2) phytoremediation capabilities under Chromium (Cr) and Lead stress (Pb). (3) To recommend the best-adapted plant species for phytoremediation in the arid climate.

## Materials and Methods

### Description of the study area

The total area of Cholistan is 26,000 km^2^, stretching from southern Bahawalpur of Punjab to the deserts of Nara and Thar in Sindh between latitudes 27° 42 N and 29° 45 N and longitude 69° 52E and 75° 24E, which is about 112 m above sea level. The desert of Cholistan is situated in a sub-tropical area, and its climate is arid to semi-arid. The mean annual rainfall ranges between 100 and 250 mm. The mean summer temperature is 34–38°C, and the average winter temperature is 15–20°C. The highest temperature could reach 51.6°C. Extreme temperatures and long droughts occasionally affect monsoon rainfall. These areas have very low humidity and high evaporation rates.

### Experimental site and design

The experiment was conducted in the field area of the department of Forestry, Range and Wildlife Management, the Islamia University of Bahawalpur, from September to October 2019. The grass stumps of *Typha angustifolia, Tragus roxburghii, Aeluropus logopoides*, and *Cenchrus ciliaris* were collected from the Cholistan rangeland. The plants were trimmed to the uniform size of the stump, which was 10.16 cm. The stumps were placed in well-prepared plastic pots. Four stumps were planted per pot, plants were assigned to control, and different heavy metal stress treatments.

Four treatments with three replications per species were used, making it a total of 24 planted stumps. The control plants were irrigated with 300 mL of tap water four times a week. Stumps under heavy metals treatment were similarly treated with 300 mL water containing a solution of heavy metal salts of Pb and Cr (the concentrations are shown in Table 1). Cr treatments in the form of K_2_Cr_2_O_7_ were applied at 0, 20, 40, and 80 μmol/L, whereas Pb was applied as PbCl_2_ at 0, 50, 200, and 500 μmol/L as control, low, moderate, and high-stress treatments, respectively. Cr and Pb salt concentrations are shown in Table 1.

**TABLE 1:** Treatment concentration levels of chromium (K_2_Cr_2_O_7_) and lead (PbCl_2_) salts.

Stress level	K_2_Cr_2_O_7_ (mg/L)		PbCl_2_ (mg/L)	
Control	T1	0	T1	0
Low stress	T2	113.15	T2	67.14
Moderate stress	T3	226.28	T3	268.15
High stress	T4	452.56	T4	536.28

The experimental design was randomized to prevent the effects of microclimatic variations, and all the pots were rotated during the experiment. The experiment was carried out under normal atmospheric conditions with average temperature and humidity were 31.2 ± 2°C and 50 ± 4%, respectively. The Plants were harvested after 6 weeks of heavy metals stress.

### Analysis of morphological parameters

After harvesting, samples were divided into two parts; one was kept for morphological parameters analysis, and the other was stored to analyze the physiological parameters. The morphological parameters such as leaves count, root and shoot length, and fresh and dry biomass were evaluated for *T. angustifolia, T. roxburghii, A. logopoides*, and *C. ciliaris* grass species under Cr and Pb stress. The root and shoot lengths were measured with a scale. Fresh biomass was measured immediately after harvesting. Representative samples of all the replicates were obtained for dry weight measurement. Dry weight was determined after oven-drying at 80°C until constant weight in the lab. The electrical balance was used to take the fresh and dry weight of the samples. Values were tabulated as a mean of three biological replicates for all the parameters.

### Physiological parameters

Physiological parameters were calculated, including chlorophyll content, membrane stability index (MSI), and leaf gas exchange.

### Chlorophyll content and membrane stability index

Chlorophyll contents were measured using the chlorophyll meter’s pad value. For MSI, one gram of fresh sample has been taken from pre-washed and autoclaved test tubes. In test tubes, 10 mL of ultrapure distilled water was applied and incubated at 45°C for 35 min. Incubation was done in the water bath, which was preheated. Electrical conductivity (C1) was calculated via a conductivity meter after incubation. A similar experiment was repeated for 15 min at 100°C. The second conductivity was measured as (C2). MSI was calculated using the formula below;


MSI=[1-(C/1C)2]×100

#### Leaf gas exchange measurements

For gas exchange parameters like net CO_2_ assimilation rate (μmol m^–2^ s^–1^), net photosynthetic rate (A), transpiration rate (mmolm^–2^ s^–1^), and stomatal conductance (mmol s^–2^ s^–1^), a portable infrared gas exchange analyzer was used. Well-lit leaves from 10 plants were carefully selected and placed in a 6 cm_3_ leaf chamber. The leaf chamber temperature was set at 20°C during the test, and the relative humidity was set to 400 μmol^–1^ as well as the CO_2_ reference. All measurements have taken place between 10:00 a.m. and 2:00 p.m. with the maximum light availability. As a combination of net CO_2_ assimilation rate and transpiration rate, effective water usage was determined (WUE) (Rasheed et al., 2015). All gas-exchange measurements were carried out at a leaf temperature of 25 ± 0.1°C, a leaf to air vapor pressure deficit of < 1.5 kPa, and a constant gas flow rate of 500 μmol s^–1^. To decrease the gas-exchange measurements time, the ambient CO_2_ concentration was increased to no more than 700 μmol mol^–1^.

### Determination of chromium and lead content

A subset of 10 plants was randomly selected from the control and heavy metal treatment. Samples were washed and air-dried at 105°C for 24 h in fluidized bed dryer. The dried matter was then pulverized with Mortimer and pestle. Pyrex digestive pipes 0.5 g of powdered plant specimens were weighed and transferred to 100 mL (10 mL (HNO_3_; HClO_4_; 7:3 ratio) and 1,800°C heated digestive mixture). The temperature slowly rose until all HNO_3_ signs vanished. White flumes were exposed. Digestion lasted 30 min. Upon digestion, the tube rack was removed from the warm plate and cooled for 2 h. The resulting plant solutions were then diluted with distilled water up to 100 mL, and each diluted solution was filtered using Watman-42 filter paper and stored in sampling bottles (Guo et al., 2015). A blank solution (containing all reagents except plant samples) was also digested with other samples. The heavy metals concentration was calculated through the atomic absorption spectrophotometer (UV-Visible Spectrophotometer Carry-60) with 4 standards using distilled water with concentration ranges of 0.5, 1.0, 1.5, 2.0 ppm and a standard deviation of ± 0.01 mg/kg and with standard compared.

### Statistical analysis

Two-way Analysis of Variance (ANOVA) was performed at a 5% probability level to investigate the treatments and species effect for all the studied parameters measured. A Pearson correlation was used to observe the association and intensity of the parameter in different species under various treatments. Values from a completely randomized design are indicated as mean ± SD, and the significant differences were compared using *post hoc* Tukey’s HSD test. Statistical analysis was performed using (IBM SPSS Statistics for Windows, Version 21.0. Armonk, NY: IBM Corp.) and figures were created using origin software.

## Results

### Morphological parameters

**FIGURE 1:**
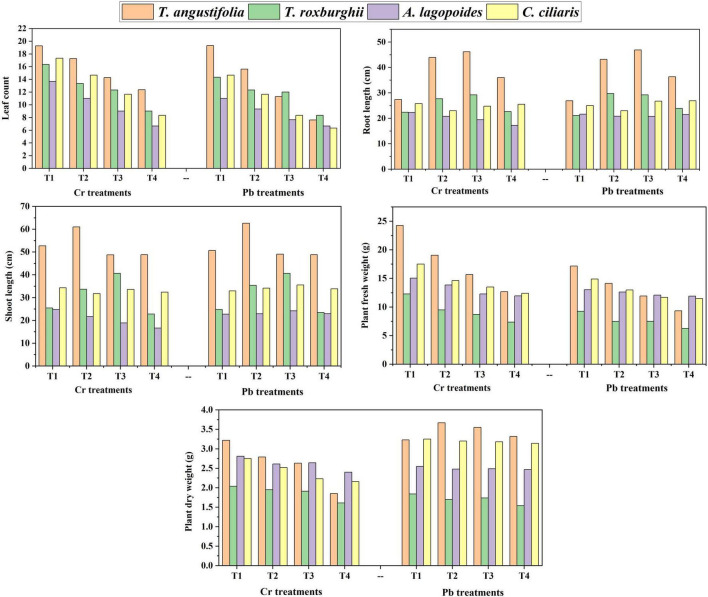
Mean values of morphological parameters (leaf count, root length, shoot length, plant fresh weight and plant dry weight) against Chromium (Cr) and Chromium (Pb) concentrations in *Typha angustifolia, Tragus roxburghii, Aeluropus lagopoides and Cenchrus ciliaris* selected grass *species*.

Columns in Figure 1 showed the values of all the morphological parameters for four grass species against Cr and Pb stresses varied significantly (Figure 1 and Supplementary Table 1). Leaf count decreased as the heavy metal stress increased in all the species. Interestingly root length and shoot length were higher under heavy metal stress conditions (T1 and T2) than in the control treatment. Plant fresh weight consistently decreased as the heavy metal stress increased in all the species. Plant dry weight also decreased as the Cr stress increased; however, no particular pattern was observed against Pb stress. Overall, *T. angustifolia* performed better, followed by *C. ciliaris*; no clear pattern was observed for *T. roxburghii* and *A. logopoides* (Figure 1 and Supplementary Table 1).

**FIGURE 2:**
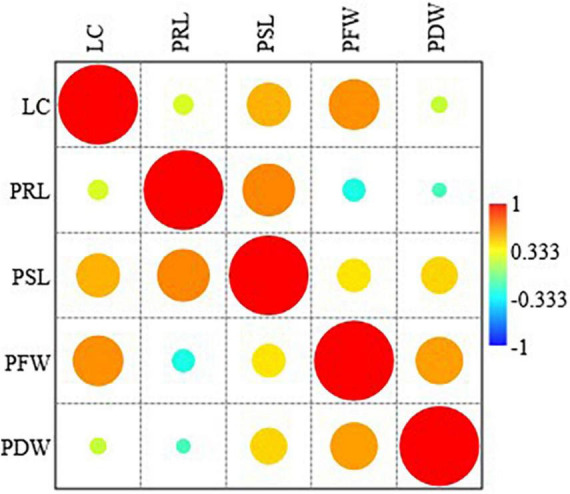
Pearson correlation analysis among the morphological parameters across all the treatments and species. LC, Leaf count; PRL, plant root length; PSL, plant shoot length; PFW, plant fresh weight and PDW, plant dry weight.

Across all grass species and treatments, leaf count was significantly correlated to shoot length and fresh biomass production. Root length was correlated to shoot length. Shoot length was also associated with fresh and dry biomass production. Moreover, there was a positive relationship between fresh and dry biomass production (Figure 2).

### Gas exchange measurements

CO_2_ assimilation rate (Co_2_d) was higher under Cr stress conditions than in the control treatment. Although no massive difference was observed, the Co_2_d increased as the Cr stress increased in all species. Co_2_d was observed to be slightly higher than *T. angustifolia* followed by *T. roxburghii*. All other gas exchange parameters, such as relative humidity (RHd), net photosynthetic rate (A), transpiration rate (E), stomatal conductance (Gs) and leaf internal CO_2_ concentration (Ci), were higher under control treatment, and gradually decreased as the Cr stress levels increased among all the species (Table 2 and Supplementary Table 3). In comparing species, all the gaseous exchange parameters under Cr stress were higher in *C. ciliaris* followed by *T. angustifolia*, *T. roxburghii* and *A. lagopoides* (Table 2 and Supplementary Table 2).

**TABLE 2:** Leaf gas exchange measurements against chromium (Cr) concentrations in *Typha angustifolia, Tragus roxburghii, Aeluropus lagopoides, and Cenchrus ciliaris* grass species.

Treatment	Co_2_d (mol m^–2^ s^–1^)	RHd	A (μmol CO_2_ m^–2^ s^–1^)	E (mmol H_2_O m^–2^s^–1^)	Gs (mmol H_2_O m^–2^ s^–1^)	Ci (ppm)
***Typha angustifolia***						
T1	12.59 ± 0.15	2.87 ± 0.02	4.24 ± 0.07	2.52 ± 0.13	23.22 ± 0.52	666.3 ± 14.52
T2	19.14 ± 0.11	2.55 ± 0.14	3.91 ± 0.13	2.25 ± 0.05	24.05 ± 0.03	615.0 ± 6.74
T3	19.55 ± 3.31	2.16 ± 0.11	3.20 ± 0.06	2.17 ± 0.05	21.74 ± 0.07	596.0 ± 7.21
T4	19.56 ± 0.29	1.93 ± 0.04	3.14 ± 0.03	2.17 ± 0.04	20.06 ± 0.07	550.6 ± 3.51
***Tragus roxburghii***						
T1	12.55 ± 0.46	2.79 ± 0.15	4.29 ± 0.01	2.33 ± 0.01	23.27 ± 0.11	679.3 ± 3.78
T2	18.69 ± 0.68	2.67 ± 0.08	3.98 ± 0.02	2.23 ± 0.01	22.98 ± 0.11	656.6 ± 6.65
T3	18.76 ± 1.74	2.55 ± 0.08	3.27 ± 0.23	2.18 ± 3.46	21.18 ± 0.69	633.6 ± 6.42
T4	18.85 ± 0.02	2.08 ± 0.09	2.87 ± 0.11	2.15 ± 0.01	19.88 ± 0.18	582.3 ± 14.64
***Aeluropus lagopoides***						
T1	14.07 ± 0.94	2.75 ± 0.05	4.71 ± 0.06	2.26 ± 0.05	23.86 ± 0.10	663.3 ± 8.32
T2	18.23 ± 0.01	2.60 ± 0.08	4.22 ± 0.23	2.19 ± 1.52	23.62 ± 0.15	655.3 ± 5.50
T3	17.53 ± 0.24	2.45 ± 0.05	3.79 ± 0.31	2.17 ± 1.21	21.92 ± 0.23	629.6 ± 17.03
T4	19.03 ± 0.28	2.29 ± 0.01	3.13 ± 0.13	2.16 ± 0.03	20.50 ± 0.48	601.0 ± 2.64
***Cenchrus ciliaris***						
T1	12.50 ± 0.44	2.76 ± 0.77	4.35 ± 0.08	2.26 ± 0.15	23.28 ± 0.14	676.0 ± 4.58
T2	17.76 ± 0.11	2.66 ± 0.02	4.26 ± 0.08	2.17 ± 0.77	22.87 ± 0.08	650.6 ± 1.15
T3	17.82 ± 0.16	2.57 ± 0.03	3.24 ± 0.06	2.20 ± 0.05	22.62 ± 0.04	640.6 ± 6.02
T4	17.84 ± 0.12	2.45 ± 0.02	3.14 ± 0.15	2.55 ± 0.20	21.83 ± 0.14	594.0 ± 12.76

Values are mean ± SD. “Co_2_d” is the CO_2_ assimilation rate, “RHd” is relative humidity, “A” is net photosynthetic rate, “E” is transpiration rate, “Gs” is stomatal conductance, and “Ci” is leaf internal CO_2_ concentration.

Regarding Pb stress, Co_2_d was higher under Pb stress conditions than in the control treatment. Significant differences were observed among species, and treatment and Co_2_d increased as the Pb stress increased among all species. All other gas exchange parameters gradually decreased as the Pb stress levels increased among all the species. While comparing species, all the gaseous exchange parameters under Pb stress were higher in *T. roxburghii* followed by *C. ciliaris* (Table 3 and Supplementary Table 2).

**TABLE 3:** Leaf gas exchange measurements against lead (Pb) Concentrations in *Typha angustifolia, Tragus roxburghii, Aeluropus lagopoides, and Cenchrus ciliaris* grass species.

Treatment	Co_2_d (mol m^–2^ s^–1^)	RHd	A (μmol CO_2_ m^–2^ s^–1^)	E (mmol H_2_O m^–2^s^–1^)	Gs (mmol H_2_O m^–2^ s^–1^)	Ci (ppm)
***Typha angustifolia***						
T1	9.48 ± 0.03	2.63 ± 0.02	4.17 ± 0.01	2.22 ± 0.08	22.3 ± 0.13	644.3 ± 17.95
T2	16.21 ± 0.07	2.26 ± 0.05	3.77 ± 0.20	2.13 ± 0.01	26.88 ± 3.18	610.3 ± 4.04
T3	17.04 ± 0.06	1.86 ± 0.05	3.12 ± 0.96	2.11 ± 0.01	20.74 ± 0.07	589.3 ± 9.01
T4	19.81 ± 0.60	1.65 ± 0.01	2.77 ± 0.17	2.07 ± 0.04	19.06 ± 0.07	548.6 ± 1.52
***Tragus roxburghii***						
T1	9.64 ± 0.18	2.70 ± 0.01	4.29 ± 0.01	2.23 ± 0.01	23.18 ± 0.01	662.6 ± 2.08
T2	12.88 ± 0.50	2.54 ± 0.77	3.94 ± 0.05	2.13 ± 0.01	22.98 ± 0.01	649.6 ± 1.52
T3	14.14 ± 0.07	2.40 ± 0.01	3.18 ± 0.01	2.08 ± 0.78	21.18 ± 0.69	621.3 ± 0.57
T4	17.23 ± 0.01	1.94 ± 0.05	2.95 ± 0.06	2.04 ± 0.05	19.91 ± 0.12	595.3 ± 3.05
***Aeluropus lagopoides***						
T1	9.12 ± 0.11	2.63 ± 0.02	4.12 ± 0.04	2.20 ± 0.01	22.90 ± 0.07	652.6 ± 2.30
T2	15.21 ± 0.07	2.55 ± 0.06	3.87 ± 0.17	2.09 ± 0.64	22.73 ± 0.15	642.0 ± 2.0
T3	16.22 ± 0.04	2.40 ± 0.06	3.13 ± 0.01	2.07 ± 0.21	20.92 ± 0.23	620.3 ± 18.47
T4	18.25 ± 0.02	2.17 ± 0.66	3.03 ± 0.09	2.04 ± 0.02	19.66 ± 0.01	576.3 ± 4.04
***Cenchrus ciliaris***						
T1	9.62 ± 0.27	2.76 ± 0.07	4.26 ± 0.01	2.23 ± 0.01	23.15 ± 0.77	659.0 ± 6.55
T2	14.15 ± 0.06	2.64 ± 0.02	4.16 ± 0.08	2.17 ± 0.30	22.88 ± 0.10	649.0 ± 2.0
T3	15.10 ± 0.16	2.53 ± 0.01	3.08 ± 0.01	2.51 ± 0.39	22.66 ± 0.01	634.0 ± 4.0
T4	15.47 ± 0.42	2.44 ± 0.03	2.98 ± 0.11	2.48 ± 0.15	21.79 ± 0.15	585.0 ± 14.1

Values are mean ± SD. “Co_2_d” is the CO_2_ assimilation rate, “RHd” is relative humidity, “A” is net photosynthetic rate, “E” is transpiration rate, “Gs” is stomatal conductance, and “Ci” is leaf internal CO_2_ concentration.

### Chlorophyll content and membrane stability index

**FIGURE 3:**
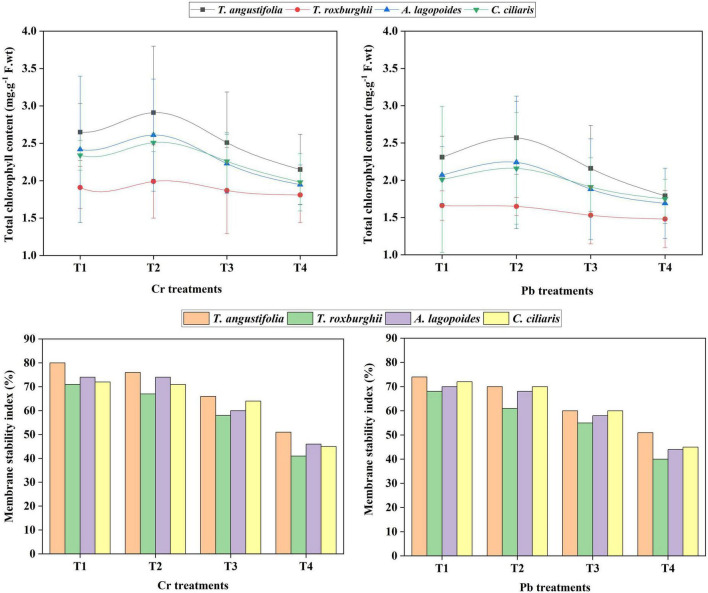
Total chlorophyll contents measurements and membrane stability index against Chromium (Cr) and lead (Pb) Concentrations in *Typha angustifolia, Tragus roxburghii, Aeluropus lagopoides and Cenchrus ciliaris* grass species.

Significant differences were observed for total chlorophyll content among all the treatments for most of the species except for *T. roxburghii* against both heavy metals. Total chlorophyll content was in order of *T. angustifolia* > *A. lagopoides* > *C. ciliaris* > *T. roxburghii* against both heavy metals. In terms of MSI, Pb had a more sever effect on MSI compared to Cr in all the species. Overall, membrane stability index decreased as the heavy metal stress increased. *T. angustifolia* showed better MSI followed by *C. ciliaris*, *A. lagopoides* and *T. roxburghii* (Figure 3 and Supplementary Table 3).

### Water use efficiency

No significant difference was observed among all the treatments for *T. angustifolia* after applying both Cr (*P* = 0.29) and Pb (*P* = 0.42) treatments. Although significant differences were observed among treatments for the remaining species, no clear patterns were seen. Overall, WUE was higher under heavy metals stress conditions than the control for all the species for both Cr and Pb. While comparing species, the WUE was in the order of *T. angustifolia* > *A. lagopoides* > *T. roxburghii* > *C. ciliaris* under both Cr and Pb stress (Table 4 and Supplementary Table 4).

**TABLE 4:** Water use efficiency (μmolm^–2^s -/mmolm^–2^ s ^–1^) of *Typha angustifolia, Tragus roxburghii, Aeluropus lagopoides, and Cenchrus ciliaris* grass species against Chromium (Cr) Concentrations.

Treatment	Chromium (Cr) stress	Lead (Pb) stress
***Typha angustifolia***		
T1	2.77 ± 0.50	3.02 ± 1.02
T2	3.50 ± 0.01	3.13 ± 0.05
T3	3.61 ± 0.06	3.36 ± 0.10
T4	3.69 ± 0.08	3.45 ± 0.01
***Tragus roxburghii***		
T1	1.75 ± 0.06	1.87 ± 0.76
T2	2.76 ± 0.01	1.92 ± 0.04
T3	2.85 ± 0.03	1.96 ± 0.10
T4	3.16 ± 0.03	1.97 ± 0.77
***Aeluropus lagopoides***		
T1	2.15 ± 0.04	2.69 ± 0.03
T2	2.44 ± 0.03	2.76 ± 0.7
T3	2.65 ± 0.06	2.74 ± 0.03
T4	2.69 ± 0.05	2.78 ± 0.04
***Cenchrus ciliaris***		
T1	1.75 ± 0.06	1.84 ± 0.01
T2	1.96 ± 0.01	1.84 ± 0.02
T3	1.85 ± 0.03	1.77 ± 0.01
T4	2.06 ± 0.03	1.93 ± 0.03

Values are mean ± SD.

### Phytoremediation of chromium and chromium

Cr and Pb concentrations were evaluated in four grass species after 6 weeks of treatment. Statistical analyses showed that changes in Cr concentration significantly affect Cr absorption by all four grass species (Table 5). *T. angustifili, T. roxburghii, A. lagopoides*, and *C. ciliaris* revealed comparable increments in metal content compared to control. It was observed that the Cr contents of *T. angustifolia* were higher than the other three species at each level of Cr stress. In terms of species, at 80 μM, the highest Cr intake was observed in all four grass species.

**TABLE 5:** Absorption of Cr and Pb by the leaves of *Typha angustifolia, Tragus roxburghii, Aeluropus lagopoides, and Cenchrus ciliaris* selected grass species.

Treatments	*Typha angustifolia*	*Tragus roxburghii*	*Aeluropus lagopoides*	*Cenchrus ciliaris*
**Cr Concentrations**				
T1	0.0024 ± 0.09	0.0018 ± 0.04	0.0211 ± 0.02	0.0012 ± 0.03
T2	0.0109 ± 0.09	0.0090 ± 0.06	0.0114 ± 0.07	0.0096 ± 0.07
T3	0.0111 ± 0.08	0.0093 ± 0.02	0.0102 ± 0.04	0.0081 ± 0.05
T4	0.0121 ± 0.05	0.0096 ± 0.05	0.0103 ± 0.06	0.0083 ± 0.05
**Pb Concentrations**				
T1	0.0023 ± 0.05	0.0016 ± 0.15	0.0020 ± 0.72	0.0151 ± 0.08
T2	0.0146 ± 0.08	0.0141 ± 0.01	0.0141 ± 0.03	0.0136 ± 0.52
T3	0.0149 ± 0.05	0.0142 ± 0.08	0.0143 ± 0.04	0.0138 ± 0.51
T4	0.0152 ± 1.52	0.0143 ± 1.38	0.0145 ± 0.05	0.0139 ± 0.07

Values are mean ± SD.

Statistical analyses revealed a highly significant effect of Pb stress on Pb uptake among all species (Table 5). As the Pb stress increased, *T. angustifolia* exhibited more absorption in Pb than *Aeluropus lagopoides, Tragus roxburghii*, and *Cenchrus ciliaris*, respectively. Like Cr, at the highest stress level of Pb, all the grass species showed the highest uptake (Table 5).

### Association between the studied parameters

**FIGURE 4:**
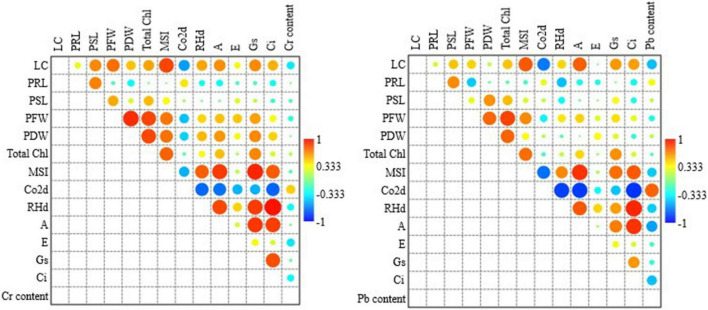
Pearson correlation analysis among all the studied parameters across all the treatments and species against Cr (left side figure) and Pb (right side figure) concentrations. LC, Leaf count; PRL, plant root length; PSL, plant shoot length; PFW, plant fresh weight; PDW, plant dry weight; Chl content, chlorophyll content; MSI, membrane sustainability index; CO_2_d, CO_2_ assimilation rate; RHd, relative humidity; A, net photosynthetic rate; E, transpiration rate; Gs, is stomatal conductance; and Ci, is leaf internal CO_2_ concentration.

Across all grass species and treatments of Cr concentrations, total chlorophyll content and MSI were strongly correlated to leaf count and fresh and dry biomass. CO_2_ assimilation rate was negatively correlated to leaf count and MSI. Net photosynthetic rate was positively correlated to MSI, RHd, leaf count, total chlorophyll content and dry biomass, where it was negatively correlated to CO_2_ assimilation rate. Stomatal conductance and leaf internal CO_2_ concentration were positively correlated to leaf count, biomass, MSI, RHd, photosynthetic rate and whereas negatively correlated to CO_2_ assimilation rate; Moreover, Stomatal conductance and leaf internal CO_2_ concentration were also correlated to each other. Apart from a negative correlation with CO_2_ assimilation rate, there was no association found for transpiration rate with any other parameter (Figure 4).

**FIGURE 5:**
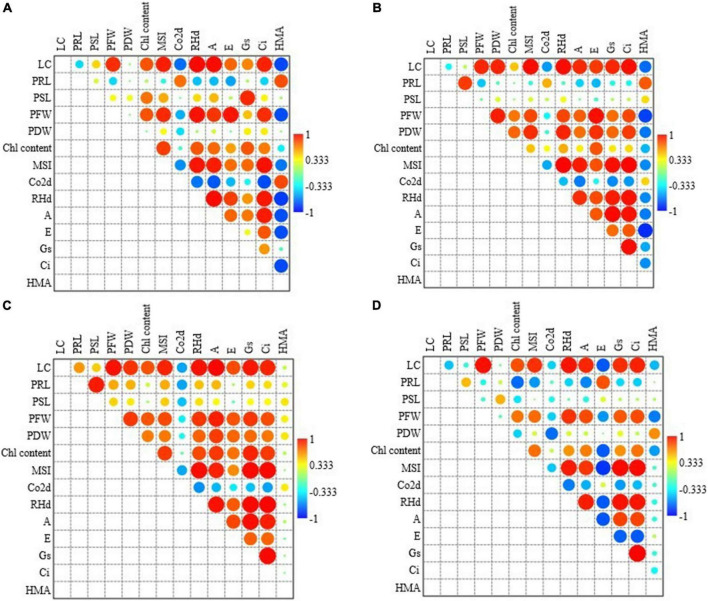
Pearson correlation analysis for **(A)** *Typha angustifolia*, **(B)** *Tragus roxburghii*, **(C)** *Aeluropus lagopoides*, and **(D)** *Cenchrus ciliaris* grass species among all the studied parameters across both heavy metals concentrations. LC, Leaf count; PRL, plant root length; PSL, plant shoot length; PFW, plant fresh weight; PDW, plant dry weight; Chl content, chlorophyll content; MSI, membrane sustainability index; CO_2_d, CO_2_ assimilation rate; RHd, relative humidity; A, net photosynthetic rate; E, transpiration rate; Gs, is stomatal conductance; and Ci, is leaf internal CO_2_ concentration.

Across all grass species and treatments of Pb concentrations, total chlorophyll content was strongly correlated to fresh and dry biomass. MSI was correlated leaf count, fresh biomass and total. CO_2_ assimilation rate was negatively correlated to leaf count and MSI. Net photosynthetic rate was positively correlated to leaf count, MSI and RHd whereas it was negatively correlated to CO_2_ assimilation rate. Stomatal conductance has a weak positive association with leaf count, total chlorophyll content, correlated to leaf count, MSI, CO_2_ assimilation rate and photosynthetic rate. Leaf internal CO_2_ concentration had a strong positive association with MSI, RHd and photosynthetic rate and a negative association with CO_2_ assimilation rate. There was no association found for transpiration rate with any other parameter. Pb absorption was positively associated with MSI and negatively correlated to leaf count, photosynthetic rate, and internal CO_2_ concentration (Figure 4). Species-wise correlation is shown in Figure 5.

## Discussion

Due to industrial wastewater, heavy metals pollution in Pakistan is worsening, negatively impacting the region’s overall biodiversity (Arooj et al., 2018; Hussain et al., 2018). Heavy metal pollution leads to the injection of toxic material into the food chain (Kadam et al., 2018). In this study, grass species showed varied growth behavior against the different concentrations of heavy metals; generally, their growth decreased with the elevated heavy metals concentrations.

Morphological and physiological parameters are considered good indicators for estimating plant tolerance against heavy metal and nutrient deficiency stresses (Jiang et al., 2015; Rashid et al., 2020a,b; Faiz et al., 2022). In this study, root and shoot lengths under low and moderate Cr and Pb stress either increased or did not show a big change compared to control and high stress; however, leaf count and biomass production decreased in all species. Based on the high toxicity of Cr and Pb heavy metals, their accumulation could lead to soil contamination. Once the soil gets contaminated, it will affect crop growth, production and yield quality. Our results support Ho et al. (2013), who stated that under excessive heavy metal stress, *Lycopersicon esculentum* showed no effect in root and shoot length; however, overall fruit production reduced. These results also correlate with Rév et al. (2017), who reported decreased root growth and shoot water content in wheatgrass cultivar Szarvasi-1 energy grass under heavy metal stress. In contrast, shoot growth and root water content were unaffected. Zhang et al. (2014) studied different morphological parameters under heavy metal (Zn, Cr, Pb, Ni) stress in king grass (*Pennisetum purpureum*) and concluded that fresh root weight was affected by increasing concentration of Cr and Pb. Similar results were observed by Yao et al. (2012), in which Pb exhibited a significant effect on fresh weight reduction (25%).

Our results contradict Richard and Weidhaas (2014) that *Sesbania drummondii* reduced root length under heavy metal stress. The possible reason is the less nutrient availability in the contaminated soil, and the roots have to grow longer to reach for nutrients under low and medium stress. However, under high stress, the root lengths were significantly reduced. Under high-stress conditions, Cr enters the root cells and interacts with nucleic acid by stimulating the aggregation of chromosomes. This ultimately resulted in the inhibition of DNA replication during cell division, leading to root growth cessation and biomass production (Uchman et al., 2017).

Heavy metals possess a toxic effect on physiological parameters in plants. Different heavy metal elements, including Cu, Cr, Cd, and Zn have been found to lower the net photosynthesis rates, WUE and stomatal conductance. In this study, apart from CO_2_ assimilation, other gaseous exchange activities (RHd, photosynthetic rate, transpiration rate, stomatal conductance and leaf internal CO_2_ concentration) were lowered with stress increase in all the species. Heavy metals cause the rate of CO_2_ production to decrease and subsequently diminish, thus reducing the leaf’s internal CO_2_ concentration, which eventually affects the gaseous exchange process, especially the photosynthetic rate. Leaf transpiration rate lessened due to reduced stomatal opening (Haider et al., 2021), which could be induced by direct/indirect interaction of heavy metals toxicity with guard cells (Rucińska-Sobkowiak, 2016). Long-term exposure to the extreme toxicity of heavy metals mostly results in water deficiency. Heavy metals considerably reduce the leaf gaseous exchange parameters, which may result in water stress due to the low water flow from the soil to the leaves (Rai et al., 2016). This reduces leaf turgor and stomatal conductance (Yadav, 2010).

Heavy metals, such as Cd, Pb and Zn, affect membrane stability by reducing cell membrane permeability (Rucińska-Sobkowiak, 2016), followed by a disturbance in the ionic balance of the cell. This leads to reduced chlorophyll concentrations (Petrovic and Krivokapic, 2020) and photosystem II activity (Rai et al., 2016), which limits photosynthesis, thus leading to metabolic disruptions (Muhammad et al., 2021). Apart from grasses and agricultural species, Chandra and Kang (2016) also presented similar results in forest tree species: gaseous exchange activities reduced with the increasing concentration of heavy metals.

Chlorophyll content in leaf tissue and membrane stability of plants is influenced by various environmental stresses such as salinity, drought, cold and heat etc. Various heavy metals (i.e., mercury (Hg), copper (Cu), Cr, cadmium (Cd), and zinc (Zn) decreased the chlorophyll content in various plant species in most cases. In this study, total chlorophyll content was higher under low stress water deficits. Moreover, increased chlorophyll contents under low stress are paired with increased CO_2_ assimilation and WUE (Wang et al., 2012; Rasheed and Delagrange, 2016).

Cadmium (Cd) and lead (Pb), among heavy metals, have become more seriously toxic to plant functions due to their widespread toxicity. The use of green plant species to treat and control wastes (metals, metalloids, salts, sewage, sludge and some xenobiotic contaminants etc.) in the water, soil, and air, has gained global importance in the new field of ecology. Grasses are considered the best tool for detoxifying heavy metals in the soil and water. In this study, *T. angustifolia* showed higher absorption of Cr than other species. Pb absorption by grasses was in the order of *T. angustifolia. A. lagopoides* > *T. roxburghii*, and *C. ciliaris.* Caldelas et al. (2012) reported that *Iris pseudacorus* L. cano remediate heavy metals from soil. Similar results were studied by Khan et al. (2021) in their review article and found that ornamental species are well adapted to absorb heavy metals from soil.

## Conclusion

The present research was used to characterize the impact of high metal stress on Cholistan grasses. Heavy metals have a significant effect on plant production that not only reduces overall production but also leads to serious damage and even causes plant mortality. *T. angustifolia* morphological attributes were higher than other species under both heavy metals. Under both Cr and Pb stress, apart from Co_2_d and WUE, other physiological parameters, including RHd, A, E, Gs, Ci, and MSI, gradually decreased as the stress increased among all the species. In contrast, total chlorophyll content was higher under low stress. WUE was in the order of *T. angustifolia* > *A. lagopoides* > *T.roxburghii* > *C. ciliaris* under both Cr and Pb stress. Cr uptake of *T. angustifolia* was higher than the other three kinds of grasses in normal growth conditions. A similar trend was observed at 20, 40, and 80 μmol/L. Increasing Pb stress levels showed significant increments in Pb absorption of all species. Local species that naturally revegetate can be much cheaper than planting/management phytoremediation. Therefore, due to better growth, increased chlorophyll level, increased CO_2_ assimilation, WUE, and higher heavy metals absorption capacity, we may infer that *T. angustifolia* could be suitable for phytoremediation in heavy metal soils.

## Data availability statement

The original contributions presented in this study are included in the article/Supplementary material, further inquiries can be directed to the corresponding author/s.

## Author contributions

TF, HB, and MRa: conceptualization. HB and RS: methodology. HB and MRi: fieldworks and chemical analysis. HB, AS, and MRi: data analysis. TF, HB, and UK: writing—original draft preparation. TF and BA: validation. TF, MRa, and BA: writing—review and editing. MRa: supervision. KQ and MJ: funding. All authors have read and agreed to the published version of the manuscript.
