# Supplementary material for: Morpho-physiological growth performance and phytoremediation capabilities of selected xerophyte grass species toward Cr and Pb stress
Source: Front Plant Sci. 2022 Sep 8;13:997120. doi: 10.3389/fpls.2022.997120 (PMC9493271; doi:10.3389/fpls.2022.997120)
Supplement: Supplementary file 1 [file Data_Sheet_1.docx]

Supplementary Material

**Table S1**. One-way ANOVA results of the studied morphological parameters against Chromium (Cr) and Lead (Pb) concentrations in *Typha angustifolia, Tragus roxburghii, Aeluropus lagopoides and Cenchrus ciliaris* grass species. LC, Leaf count; PRL, plant root length; PSL, plant shoot length; PFW, plant fresh weight and PDW, plant dry weight.

|  | Species | | Leaf count | | PRL (cm) | | PSL (cm) | | PFW (g) | | PDW (g) | |
| --- | --- | --- | --- | --- | --- | --- | --- | --- | --- | --- | --- | --- |
|  |  | DOF | F | P | F | P | F | P | F | P | F | P |
|  | *T. angustifolia* | 3 | 48.1** | 0.001 | 44.3** | 0.001 | 50.2** | 0.001 | 595** | 0.001 | 83.2** | 0.001 |
| Cr | *T. roxburghii* | 3 | 54.8** | 0.001 | 85.8** | 0.001 | 267** | 0.001 | 224** | 0.001 | 32.9** | 0.001 |
|  | *A.lagopoides* | 3 | 63.7** | 0.001 | 78.0** | 0.001 | 319** | 0.001 | 608** | 0.001 | 5.48* | 0.02 |
|  | *C. sciliaris* | 3 | 54.1** | 0.001 | 9.78** | 0.004 | 15.3** | 0.001 | 32.4** | 0.0001 | 29.5** | 0.001 |
|  |  |  |  |  |  |  |  |  |  |  |  |  |
|  | *T. angustifolia* | 3 | 133** | 0.001 | 27.1** | 0.001 | 29.9** | 0.01 | 131** | 0.001 | 16.8** | 0.008 |
| Pb | *T. roxburghii* | 3 | 25.0** | 0.002 | 87.8** | 0.001 | 150** | 0.001 | 16.4** | 0.009 | 1.75 | 0.23 |
|  | *A.lagopoides* | 3 | 43.6** | 0.001 | 5.14* | 0.02 | 20.9** | 0.001 | 14.9** | 0.0012 | 1.15 | 0.38 |
|  | *C. sciliaris* | 3 | 37.4** | 0.001 | 7.96** | 0.008 | 1.12 | 0.412 | 7.85** | 0.009 | 0.81 | 0.52 |

**Table S2**. One-way ANOVA results of gas exchange parameters against Chromium (Cr) and Lead (Pb) concentrations in *Typha angustifolia, Tragus roxburghii, Aeluropus lagopoides and Cenchrus ciliaris* grass species. ‘Co_2_d’ is the CO_2_ assimilation rate, ‘RHd’ is relative humidity, ‘A’ is net photosynthetic rate, ‘E’ is transpiration rate, ‘Gs’ is stomatal conductance, and ‘Ci’ is leaf internal CO_2_ concentration.

| Species | | Co_2_d | | RHd | | A | | E | | Gs | | Ci | |
| --- | --- | --- | --- | --- | --- | --- | --- | --- | --- | --- | --- | --- | --- |
|  |  |  |  |  |  | (μmol CO_2_ m^-2^ s^-1^ ) | | (mmol H_2_O m^-2^ s^-1^ ) | | (mmol H_2_O m^-2^ s^-1^ ) | |  |  |
|  | DOF | F | P | F | P | F | P | F | P | F | P | F | P |
| Cr stress | | | | | | | | | | | | | |
| *T. angustifolia* | 3 | 12.8** | 0.002 | 58.3** | 0.0001 | 125** | 0.0001 | 12.2** | 0.002 | 3040** | 0.0001 | 87.2** | 0.0001 |
| *T. roxburghii* | 3 | 45.1** | 0.0001 | 26.1** | 0.0002 | 77.1** | 0.0001 | 136** | 0.0001 | 59.0** | 0.0001 | 65.8** | 0.0001 |
| *A.lagopoides* | 3 | 55.1** | 0.0001 | 38.2** | 0.0001 | 30.6** | 0.0001 | 7.08* | 0.01 | 91.3** | 0.0001 | 24.0** | 0.0002 |
| *C. sciliaris* | 3 | 43.5** | 0.0001 | 89.6** | 0.0001 | 148** | 0.0001 | 5.27* | 0.02 | 88.0** | 0.0001 | 63.6** | 0.0001 |
| Pb stress | | | | | | | | | | | | | |
| *T. angustifolia* | 3 | 676** | 0.0001 | 293** | 0.0001 | 66.6** | 0.0001 | 17.5** | 0.0005 | 13.3** | 0.001 | 45.5** | 0.0001 |
| *T. roxburghii* | 3 | 804** | 0.0001 | 375** | 0.0001 | 663** | 0.0001 | 161** | 0.0001 | 57.4** | 0.0001 | 664** | 0.0001 |
| *A.lagopoides* | 3 | 3831** | 0.0001 | 36.4** | 0.0001 | 80.3** | 0.0001 | 162** | 0.0001 | 333** | 0.0001 | 37.3** | 0.0001 |
| *C. sciliaris* | 3 | 43.2** | 0.0001 | 103** | 0.0001 | 432** | 0.0001 | 1.98** | 0.0191 | 117** | 0.0001 | 49.3** | 0.0001 |

**Table S3**. One-way ANOVA results for the membrane sustainability index (MSI) and Chlorophyll content against Chromium (Cr) Lead (Pb) concentrations in *Typha angustifolia, Tragus roxburghii, Aeluropus lagopoides and Cenchrus ciliaris* grass species.

| Cr | Species | | MSI | | Chlorophyll contents | |
| --- | --- | --- | --- | --- | --- | --- |
|  |  | DOF | F | P | F | P |
|  | *T. angustifolia* | 3 | 25.3** | 0.0002 | 11.0* | 0.0354 |
|  | *T. roxburghii* | 3 | 44.8** | 0.0001 | 9.47** | 0.0052 |
|  | *A.lagopoides* | 3 | 50.2** | 0.0001 | 14.8** | 0.0012 |
|  | *C. sciliaris* | 3 | 78.0** | 0.0001 | 7.01 | 0.125 |
|  |  |  |  |  |  |  |
| Pb | *T. angustifolia* | 3 | 54.7** | 0.0001 | 5.19* | 0.021 |
|  | *T. roxburghii* | 3 | 32.7** | 0.0001 | 4.87* | 0.035 |
|  | *A.lagopoides* | 3 | 70.1** | 0.0001 | 25.4** | 0.002 |
|  | *C. sciliaris* | 3 | 59.2** | 0.0001 | 16.8 | 0.0802 |

**Table S4**. One-way ANOVA results for water use efficiency (WUE) against Chromium (Cr) Lead (Pb) concentrations in *Typha angustifolia, Tragus roxburghii, Aeluropus lagopoides and Cenchrus ciliaris* grass species.

| Species | | WUE against Cr stress | | WUE against Pb stress | |
| --- | --- | --- | --- | --- | --- |
|  | DOF | F | P | F | P |
| *T. angustifolia* | 3 | 1.46 | 0.29 | 1.02 | 0.432 |
| *T. roxburghii* | 3 | 21.0** | 0.0004 | 15.4** | 0.001 |
| *A.lagopoides* | 3 | 50.9** | 0.0041 | 49.5** | 0.002 |
| *C. sciliaris* | 3 | 23.3** | 0.0003 | 99.6** | 0.001 |
